# Two Rediscoveries of the Autostereogram in the 1960s

**DOI:** 10.1177/2041669520908895

**Published:** 2020-02-27

**Authors:** Tadamasa Sawada, Galina I. Rozhkova

**Affiliations:** School of Psychology, National Research University Higher School of Economics, Moscow, Russia; Institute for Information Transmission Problems (Kharkevich Institute), Russian Academy of Sciences, Moscow, Russia

**Keywords:** binocular vision, autostereogram, wall paper illusion, visual illusion

## Abstract

The autostereogram (ASG) was discovered in the 1840s and again in the 1960s. It is acknowledged that Pete Stephens rediscovered the ASG serendipitously when he constructed an image with a repetitive pattern manually in the late 1960s. But, the principle and application of the ASG were described by Lev Mogilev from Irkutsk State University earlier in the 1960s.

Vision science was often revised and replenished by new phenomena, theories, and hypotheses during the last half century. Interestingly, such *new* ideas occasionally shed light on older, unnoticed studies that reported the same or analogous phenomena (Bergua & Skrandies, 2000; Howard, 1996). The autostereogram (ASG) is one of these cases. In this brief report, we introduce some previously unknown studies of the ASG by Lev Mogilev who created images of ASGs early in the 1960s.

An ASG is a single two-dimensional image that allows viewers to perceive a depth distribution without using any kind of stereoscopic device. The ASG’s image consists of a main pattern that is horizontally repetitive with local modulations of the pattern along the horizontal direction. The ASG is designed such that the modulations produce the percept of a nonplanar depth distribution when a stereo pair of retinal images of the repetitive pattern are fused with an unusual correspondence (e.g., see Figure 1a). You will perceive a nonplanar depth distribution from this ASG when the left retinal images of columns *C*_1–5_ correspond with the right retinal images of columns *C*_2–6_, respectively. This unusual correspondence can be induced by diverging the eyes horizontally. The whole distribution then appears farther than the physical plane. The whole distribution appears closer than the plane with the depth reversal of the distribution when the left retinal images of *C*_2–6_ correspond with the right retinal images of *C*_1–5_ (induced by converging the eyes). Note that a completely regular repetition would prompt a purely planar percept, nearer, or farther than the physical plane according to the types of correspondence induced by converging or diverging the eyes (see Kohly & Ono, 2002; Logvinenko et al., 2001; Logvinenko & Steinman, 2002, for a discussion about the mechanisms underlying this phenomenon). This illusory percept is called the *wallpaper illusion*. It was reported in 1738 by Smith and in 1844 by Brewster (Howard & Rogers, 2012; Tyler, 2014).

**Figure 1.**
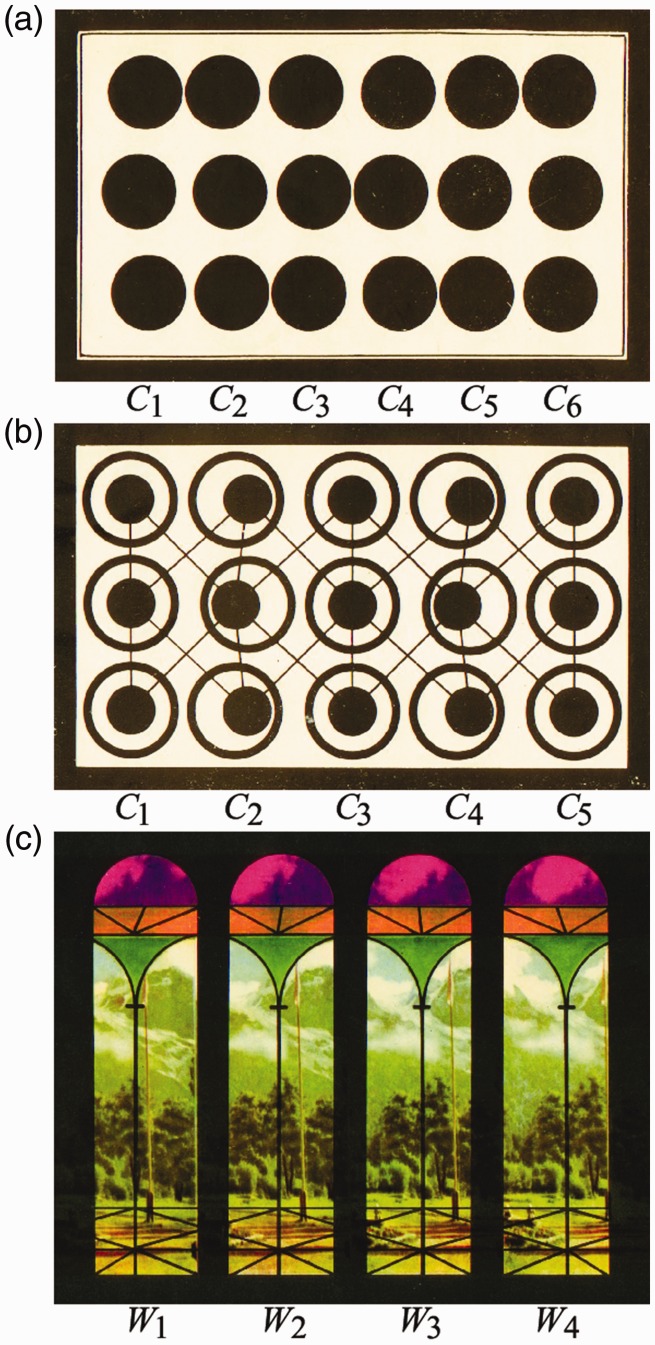
Autostereograms in Mogilev (1963).

The ASG can be regarded as derivative from the wallpaper illusion first reported by Brewster (1844), who realized local misalignments in the repetitive pattern of the wallpaper would produce perceived depth structure relative to the planar percept of the regular repetition. Such ASGs were subsequently rediscovered serendipitously in the late 1960s by Pete Stephens in California (Tyler, 2014). There are, however, earlier examples of ASGs in the magazine *Angara* (Mogilev, 1963, Figure 1). These ASGs were generated by Lev N. Mogilev, a biologist (Figure 2). This observation of ASGs was also described in his earlier studies (Mogilev, 1961, 1962), but their images were not illustrated (see Figure 3).

**Figure 2.**
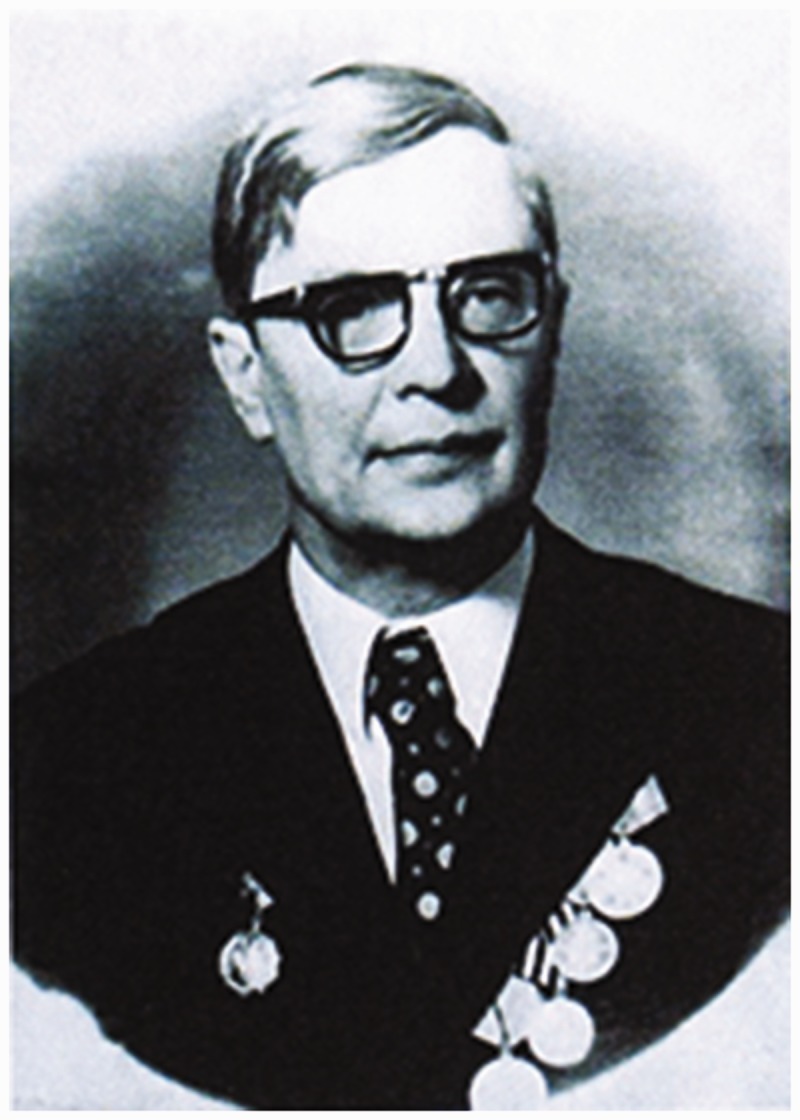
Lev N. Mogilev (1922–1985). Born in Irkutsk, Russia, in 1922. He graduated from Irkutsk State University (ISU), Department of Biology, in 1949 and defended his thesis for the Candidate-of-Sciences degree in zoology at ISU in 1955. Mogilev had founded the Department of Human and Animal Physiology at ISU and headed this department between 1969 and 1985. In 1979, he defended his thesis *Spatial visual effects as the indicators of functional organization of the visual centers* for the DSc degree. Mogilev was also known as a science fiction writer, poet, and painter (see Karatsup & Milgunov, 2019 for more information).

**Figure 3.**
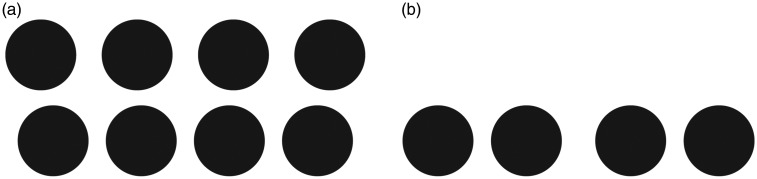
Autostereograms were reproduced by following descriptions in Mogilev (1961, 1962). (a) Two rows of four circles with different intervals. These rows are perceived with different depth positions when they are seen as an autostereogram. (b) Distance between the first and second circles and between the third and fourth circles is shorter than distance between the second and third circles. A nonplanar depth distribution is perceived when they are seen as the autostereograms.

Mogilev (1961, 1962) studied how the wallpaper illusion is affected by the positions, colors, sizes, and shapes of the elements composing a pattern. He reported that the perceived depth positions of the elements change depending on horizontal translations of the elements in the pattern. In his later monograph (Mogilev, 1982), he also reported that perceived three-dimensional (3D) orientations of the elements are affected by horizontal scaling and horizontal shear of the elements and the perceived depth changes continuously.^1^ This 3D orientation effect can be observed in the ASGs in 1963 (Figure 1b and c) but is not described there.

These independent rediscoveries of the ASG by Stephens and Mogilev in the 1960s are not surprising. There was a growing interest in 3D movies in the 1950s (Mayorov, 2014; Zone, 2012), and Julesz (1960) published his first article with random-dot stereograms. People were very interested in stereo 3D perception, and knowledge about stereo perception was being accumulated in that period. It seems that the time was ripe for the ASG to be rediscovered when it was.

## Note

1 Horizontal scaling was also tested in Mogilev (1961, 1962), but he did not report the 3D orientation effect.
